# Face validity of a new mixed-reality simulator for training endoscopic submucosal dissection: a Western pilot study

**DOI:** 10.1016/j.igie.2025.10.018

**Published:** 2025-10-31

**Authors:** Henriette Heinrich, Pieter Dewint, Phillipe Leclercq, Jelle Haringsma

**Affiliations:** 1 Universitäres Bauchzentrum Clarunis, University of Basel, Basel, Switzerland; 2 Gastroenterology and Hepatology, UZA, Antwerp, Belgium; 3 Department of Gastroenterology, CHC-MontLegia, Liège, Belgium; 4 University Hospitals Leuven, Leuven, Belgium; 5 Gastroenterology and Hepatology, Meander Medical Center, Amersfoort, the Netherlands; 6 Gastroenterology and Hepatology, Meander Medical Center, Amersfoort, The Netherlands

## Abstract

**Background and Aims:**

Endoscopic submucosal dissection (ESD) is an effective but technically demanding procedure with a steep learning curve. Traditional training methods, including master courses, apprenticeship, and animal models, have ethical, financial, and logistical constraints. This pilot study evaluates the face validity of a novel mixed-reality simulator designed to enhance and maintain endoscopic dexterity in ESD training.

**Methods:**

In this exploratory, prospective observational cohort study, 8 ESD trainees with prior advanced endoscopy experience, but limited ESD exposure, underwent a 2-day structured training using the EndoCubot (EndoRobotics, Seoul, South Korea) simulator with artificial laminated tissue (VTT [Versatile Training Tissue], KOTOBUKI Medical, Yashio, Japan). Performance was assessed pre- and post-training through a 5-minute dexterity test simulating circular incision with 40 achievable points, and a questionnaire evaluating simulator realism, satisfaction, and training effectiveness. Paired sample *t* tests (α = 0.05) and descriptive statistics were used for analysis. Results were reported as mean with standard deviation.

**Results:**

On a 40-point scale, mean postcourse scores (34.7 [4.86]) were significantly higher than precourse scores (25.50 [0.76], *P* < .001), with a mean improvement of 9.25 points (95% confidence interval, 4.993-13.507, Hedges’ *g* correction = 1.613). Survey results indicated high satisfaction (mean rating: 9.125/10) and strong agreement on the simulator's realism and effectiveness.

**Conclusions:**

The simulator demonstrates strong face validity and supports a realistic standardized skill acquisition in ESD. The simulator could provide a realistic and ethical alternative to current animal models and requires further study.

## Introduction

Endoscopic submucosal dissection (ESD) is an advanced minimally invasive procedure to remove early-stage tumors from the superficial layers of the gastrointestinal tract. The technique enables an en bloc resection with accurate pathology staging. It is a tedious, technically demanding procedure and requires extensive knowledge, specialized equipment, and a high level of new endoscopic skill. Even for experienced endoscopists, ESD is characterized by a steep learning curve and possible loss of dexterity due to lack of continuous practice. Western ESD training methods often rely on hands-on practice using explant models and live porcine models.^1^ These models are limited by availability, ethical concerns, costs, anatomical discrepancies, and logistical hurdles related to specimen procurement, maintenance, and endoscope hygiene.^2^, ^3^, ^4^

Over the last decades, multiple endoscopy training simulators have been constructed and evaluated.^2^^,^^3^ These simulators offer a risk-free, reproducible environment for repetitive practice, allowing trainees to hone their skills before performing procedures on patients. Several studies have demonstrated that computer-assisted models (eg, virtual reality platforms) can effectively supplement conventional training by accelerating skill acquisition and enhancing procedural competency.^3^, ^4^, ^5^ Moreover, development of mixed-reality models (ie, simulators that blend real-world and virtual elements) using artificial tissue have improved the realism and educational value of simulation-based training.^6^

The EndoCubot simulator (EndoRobotics, Seoul, South Korea) first introduced in 2024 represents a significant advancement by bringing everyday real instruments into a virtual reality platform with a dynamic positioning system that mimics gastrointestinal conditions. Unlike conventional models, EndoCubot enables precise manipulation of standard endoscopic instruments while simulating different anatomical lesion positions, physiological movements (eg, respiration and peristalsis), and distinct lesion architecture. By eliminating the need for live animal models, it addresses ethical concerns, reduces training costs, and might assist in ensuring standardized skill acquisition and dexterity improvement.

To our knowledge, this is the first pilot study aimed to assess the content and face validity of the EndoCubot simulator as a novel training tool for ESD, evaluating its impact on endoscopic dexterity.

## Materials and methods

### EndoCubot simulator

The EndoCubot is an innovative mixed-reality positioning simulator specifically designed for ESD training (Fig. 1). Departing from traditional reliance on animal or ex vivo specimens, it offers a controlled, reproducible training environment that closely mimics various clinical endoscopic scenarios. The simulator uses artificial laminated tissue (VTT [Versatile Training Tissue]; KOTOBUKI Medical, Yashio, Japan) to replicate the architecture of the gastrointestinal wall, enabling high-fidelity practice. Its features include adjustable wall positioning, simulated tissue movements (including respiratory and peristaltic motions), and dynamic feedback to improve endoscopic dexterity. The EndoCubot allows simulation of a variety of gastrointestinal anatomies ranging from the esophagus, stomach, and colorectum but also more complex regions such as the cardia in retroflexion and gastric angle (Fig. 1C). Additionally, EndoCubot delivers objective performance metrics, facilitating data-driven assessments of trainee progression and ensuring standardized training.

**Figure 1:**
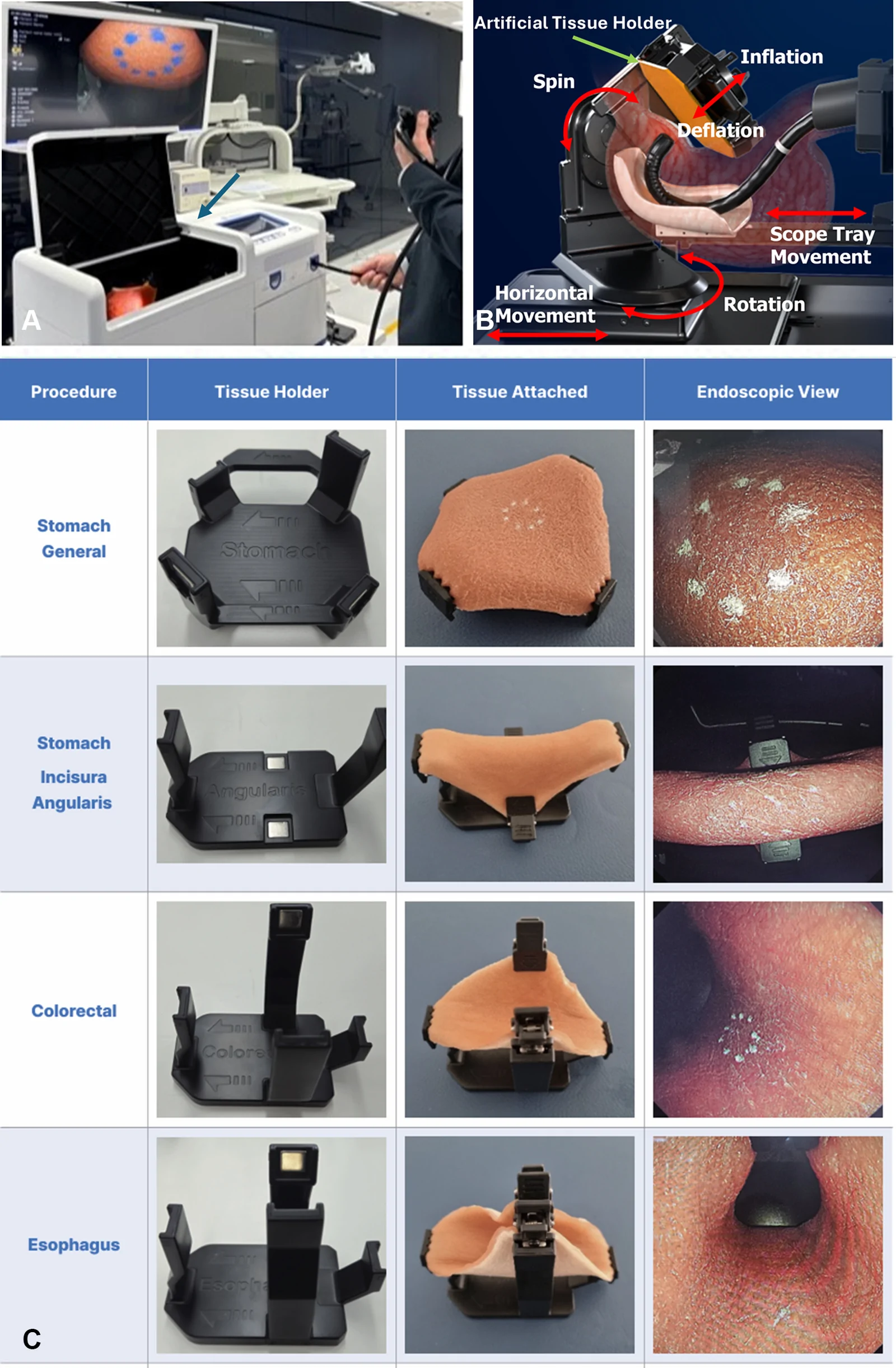
**A-C**, Endoscopic submucosal dissection training simulator from EndoRobotics (Seoul, South Korea). **A,** Overall training set-up with the EndoCubot (*arrow*) and endoscopy tower. **B,** Internal machine mechanism with scope tray and artificial tissue holder (*green arrow*). *Red arrows* show the possible movements of the scope tray and specimens. **C,** Possible anatomical settings of artificial tissue with various holder trays that get inserted into the EndoCubot for simulation of different anatomical scenarios for resection.

### Study design

A prospective observational cohort pilot study was conducted to evaluate the face validity of the EndoCubot simulator for ESD training. Improvements in endoscopic dexterity and trainee perceptions were assessed using standardized performance metrics and questionnaire-based evaluations.

### Participants

Eight ESD trainees were recruited from interventional endoscopy expert centers across Europe. Inclusion criteria required prior experience in basic upper and lower endoscopic procedures (biopsies, hemostasis, polypectomies, mucosal resections) and limited exposure to clinical ESD (<20 procedures).

### Dexterity training

The simulator-based training was delivered under the guidance of 3 experts over 2 days (minimum 2.5 hours training time per trainee per day) in February 2025 using the EndoCubot simulator with artificial laminated tissue (ie, VTT). Procedures were performed with a standard 4-mm injection needle, a standard DualKnife J (Olympus, Tokyo, Japan), and an Olympus ESG-300 electrosurgical generator with standard ESD settings. No equipment failures were observed. The curriculum used a structured, stepwise approach that progressively increased task complexity, that is, lesion and position complexity as well as tissue movements. Trainees engaged in standardized ESD exercises—including lesion marking, submucosal injection, circumferential cutting, and en bloc dissection—to build conventional ESD-specific endoscopic dexterity. The anatomic positions used for training were the greater curvature aspect of the antrum, anterior wall of the antrum, incisura angularis, gastric cardia in retroflexion, as well as proximal rectum and colon.

### Assessments and evaluation

#### Dexterity test

An identical 5-minute test using the antrum posterior wall position of the simulator was administered at both the start and conclusion of the training. The test developed for this study involves the marking and cutting of 2 circular 3-cm (1.18-inch) and 4.5-cm (1.77-inch) lesions on the VTT sample in the clockwise or counterclockwise fashion (Fig. 2). Performance within the allotted time was quantified based on the accuracy of lesion marking, where each successfully placed mark is awarded with 1 point, and completeness of circumferential cutting, where each complete connecting cut between 2 marks is awarded with 1 point. Thereby, a maximum performance score of 40 points can be obtained (Fig. 3).

**Figure 2:**
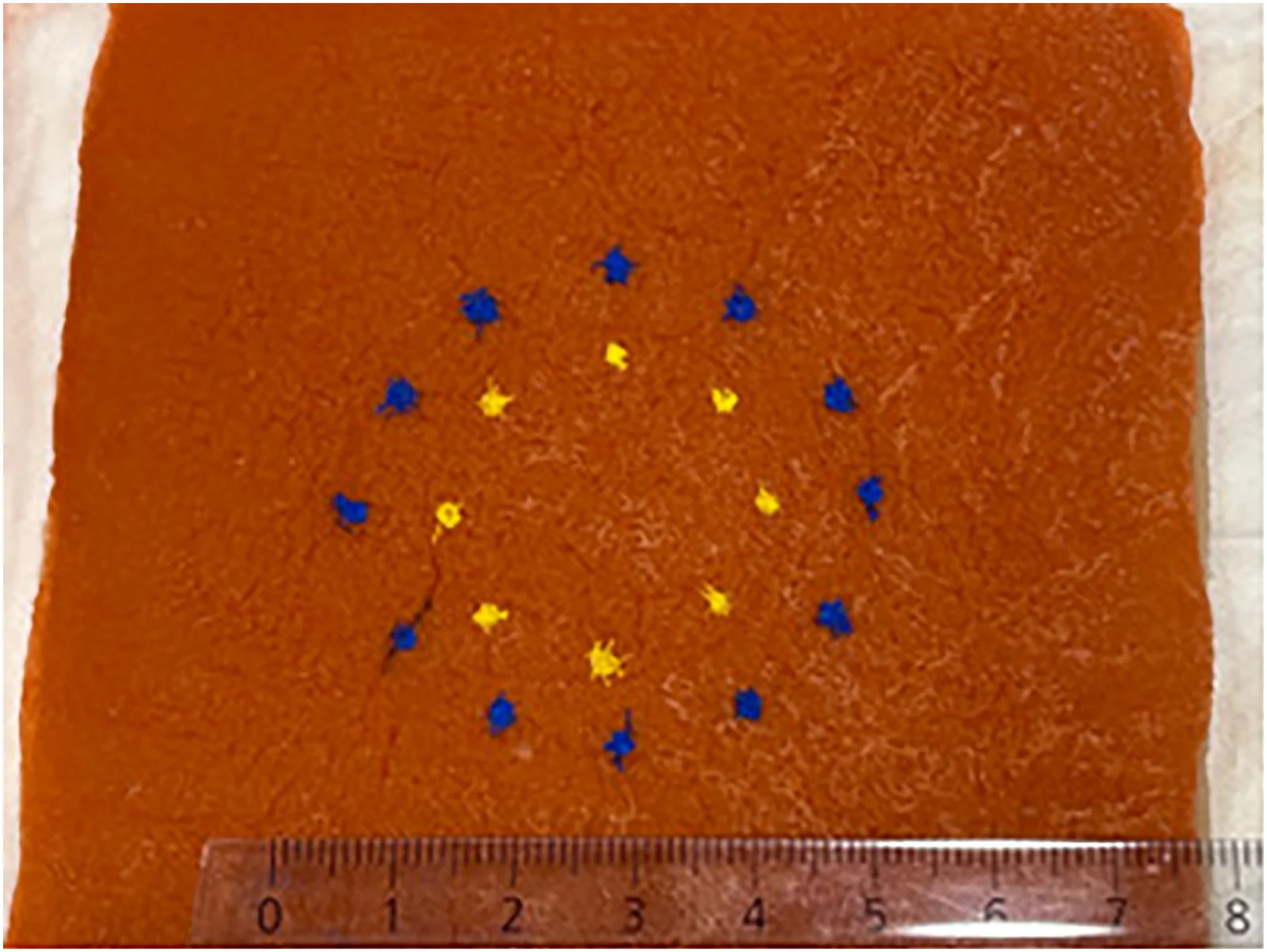
Assessment involving the marking and cutting of 2 circular lesions 3 cm (1.18 inches; *yellow*) and 4.5 cm (1.77 inches; *blue*) in diameter on the plant-based tissue.

**Figure 3:**
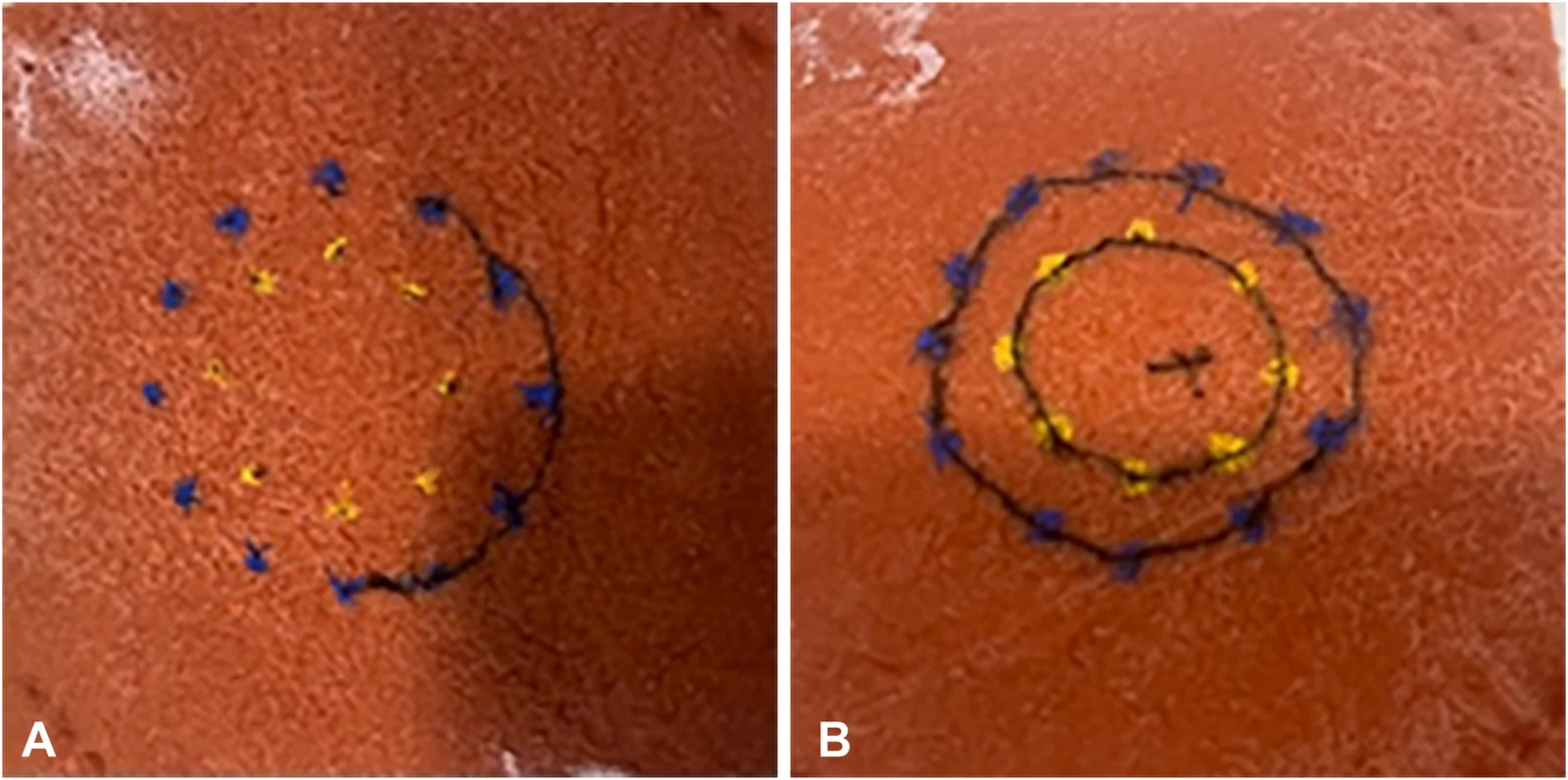
Example of the paired dexterity assessment in the antrum posterior wall position at the start with only about half of the *blue dots* connected (**A**) and conclusion of the training with all *yellow* and *blue dots* connected correctly (**B**).

#### Questionnaire-based evaluation

Post-training, validity-based evidence was obtained through a subjective survey-based analysis. The survey designed for this study included 14 statements evaluating training effectiveness, simulator performance, and comparison with porcine training models. A 5-point Likert scale was used to assess participants' perceptions of the simulator and the training program.

#### Statistical analysis

Data were analyzed using IBM SPSS Statistics version 30.0 (IBM, Armonk, NY, USA). A paired-sample *t* test was conducted to assess differences between pre- and postcourse performance scores. Effect sizes were calculated using Hedges’ *g* correction to quantify the magnitude of observed differences. Boxplot analysis was performed to visualize performance score distributions before and after training.

Descriptive statistics, including medians, ranges, interquartile ranges, and means were calculated for survey responses. All statistical analyses were performed 2-sided using a significance threshold of α = 0.05.

Results were reported as mean (strandard deviation [SD]), median (interquartile range), and 95% confidence interval.

#### Ethical considerations

All participants were assured that study data would remain anonymous, confidential, and be managed in strict accordance with the protocol. No data were shared with third parties or contracting authorities.

## Results

### Participant demographics

The study sample consisted of 8 participants (2 females and 6 males) from 8 European expert centers, all of whom were ESD novices but had finished endoscopic training in their country of origin. On average, participants had attended 1.6 prior ESD training courses (SD 1; range: 0-3) and had performed an average of 7.8 ESD procedures (SD 7; range: 0-20).

### Performance score outcomes

The mean postcourse score of 34.7 (4.86), of a maximum score of 40 points, was significantly higher than the precourse score of 25.50 (0.76) (*P* < .001). Analysis yielded a mean difference of 9.25 points, with the 95% confidence interval ranging from 4.99 to 13.51 points (Fig. 4). Effect size calculations with a Hedges’ *g* correction at 1.613 further supported this meaningful improvement in performance.

**Figure 4:**
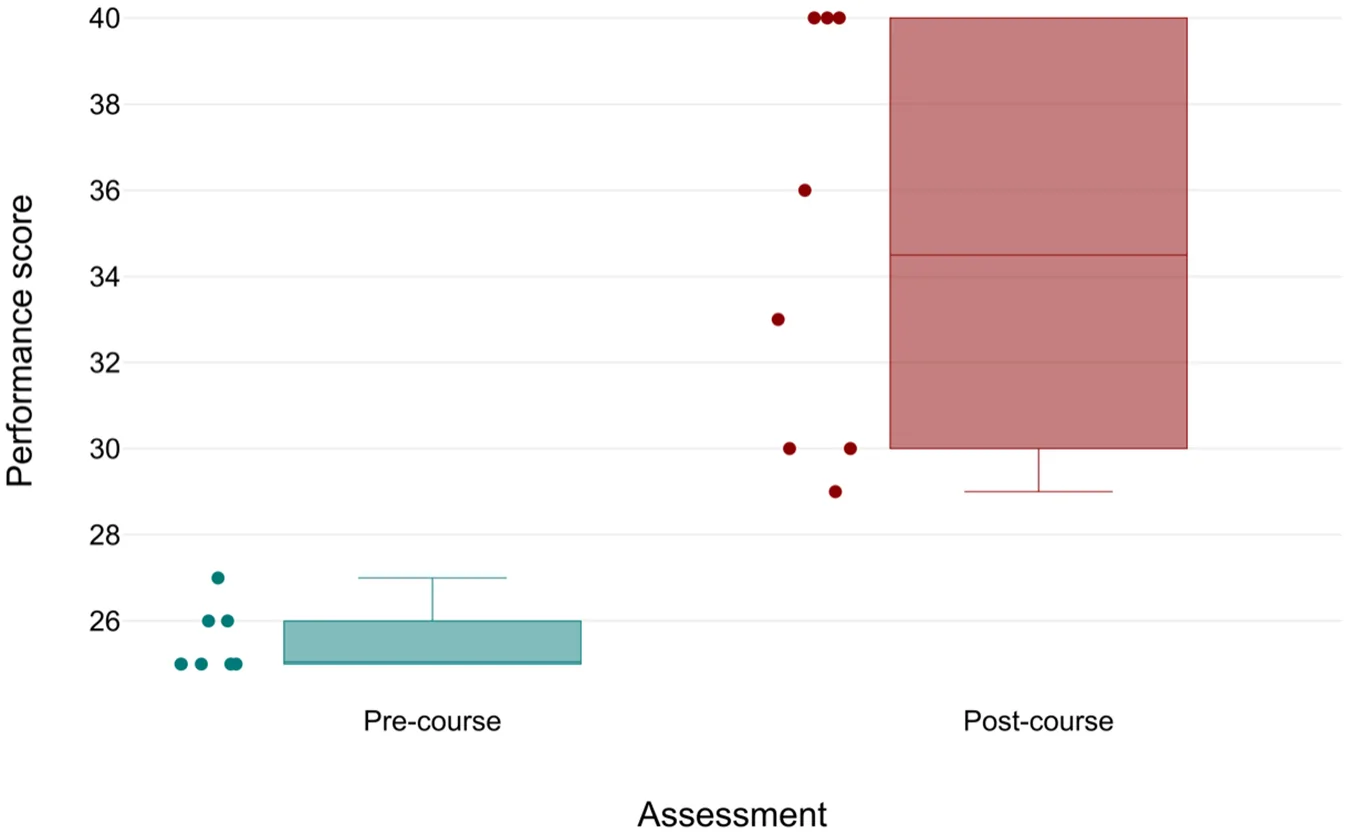
Boxplot of pre- and postcourse performance score results expressed as mean (standard deviation): 25.50 (0.76) versus 34.75 (4.86), *P* < .001.

### Survey findings

Subjective evaluations of the EndoCubot simulator and training course were collected using a 5-point Likert scale. The EndoCubot simulator received high marks. Analysis of the questionnaire responses showed that most of the 14 statements regarding training and simulator performance rated at the upper end of the scale (medians of 4-5) (Table 1). Specific aspects of the training program—including the quality of the learning environment, appropriateness of the time allocated on the simulator, and the usefulness of instructor feedback—received consistently high ratings. Similarly, items related to the technical functionality of the simulator (eg, tissue simulation quality, realistic training scenario simulation, and wall movement representation) were also rated highly. Furthermore, participants expressed a preference for this simulator over traditional animal-based models, with positive responses regarding its utility.

**Table 1:** Post-training evaluation statements grouped by topic and rated on a Likert scale

Evaluation statement	Median score∗	Min/Max	IQR	Mean
Training program				
Learning environment was satisfactory	5	4/5	0	4.86
Time allocated on the simulator was satisfactory	4	4/5	1	4.43
Training feedback by instructors	5	4/5	1	4.71
Presence of other trainees was not hindering	5	4/5	1	4.57
Observing other trainees perform was helpful	5	4/5	0	4.86
EndcoCubot simulator (EndoRobotics, Seoul, South Korea)				
Simulator functioned technically well	5	4/5	1	4.71
Simulator in this training is useful	5	4/5	1	4.71
Quality of tissue simulation	4	3/5	1	4.29
Simulates real-life training scenarios	5	3/5	1	4.43
Simulated wall movements are helpful	4	4/5	1	4.43
I would like more ESD training with simulators	5	4/5	1	4.57
Simulator in comparison				
Simulator useful addition to training on patients	5	4/5	1	4.71
Simulator is better than the pig stomach model	4	3/5	2	4.00
Simulator is preferred over live pigs	4	3/5	2	4.14

*ESD*, Endoscopic submucosal dissection, *IQR*, interquartile range; *Min*, Minimum score given; *Max*, maximum score given.

∗ Likert scale scores: 1 = strongly disagree, 2 = disagree, 3 = neutral, 4 = agree, 5 = strongly agree.

## Discussion

ESD is a technically demanding procedure that requires a high level of endoscopic skill and hands-on experience. Generally, a minimum of 20 hours hands-on practice in a learning environment is advised. Studies suggest that subsequently more than 30 supervised clinical cases are necessary to achieve proficiency and reduce adverse events.^1^ However, access to expert mentorship and structured master training programs remain limited globally, posing significant barriers to standardized ESD education.^1^^,^^7^ Furthermore, dexterity deficit and dexterity decay undermine the psychomotor component of ESD also in more experienced hands. In high-volume centers, abundant opportunities exist for effective practice and dexterity improvement; however, the average clinical environment is frequently not conducive to effective training practice.

To address this, various training models have been developed outside frontline medical practice. The most widely used are porcine models. These models have demonstrated benefit in tutored learning environments by accelerating the learning curve. Despite their relative realism, porcine models present considerable limitations such as tactile differences (eg, mucosal thickness with high tissue resistance), ethical concerns, high costs (both financially and ecologically), logistical demands (including biosafety, anesthesia, and animal handling), and access to veterinary and technical support as well as dedicated endoscopes for animal use.^8^

Virtual reality simulators, such as Endosim (Endosim USA, Bolton, Mass, USA), have demonstrated strong face validity and efficacy for basic endoscopy training but have limitations in advanced endoscopy training.^3^, ^4^, ^5^^,^^9^, ^10^, ^11^, ^12^ They lack tactile realism, including elasticity, tissue resistance, and haptic feedback, and often simulate instrument handling in oversimplified environments, limiting their suitability for ESD training and skill maintenance. Conversely, a recent study on a polyvinyl alcohol hydrogel–based ESD simulator found that repeated training sessions led to improved complete resection rates and reduced perforation rates. This supports the role of high-fidelity simulation in progressive skill development before transitioning to live procedures.^6^

The EndoCubot used in this small exploratory pilot experiment potentially addresses this gap by combining actual endoscopic instruments with a dynamic positioning system that mimics human physiology and anatomy, including folded lesions, lesion movement, tissue resistance, and variable lesion locations. Unlike animal or merely virtual models, it can provide a controlled, reproducible, and ethical environment for practicing, repeating, and assessing ESD-related skills (Video 1, available online at www.igiejournal.org).

In our pilot study, the EndoCubot demonstrated strong face validity, with a 36.3% mean improvement in performance scores after a short, structured training program. This number may underestimate the benefit, because 25% of participants completed the post-test well within the allocated time, indicating enhanced efficiency. However, in this exploratory study, we could not assess how much of this improvement is transferable to real-life ESD procedures in patients, an important limitation shared with most simulation-based studies.

Beyond objective performance, the trainees subjectively rated the simulator highly across multiple domains, including realism, usability, and perceived effectiveness. Given their background in upper and lower gastrointestinal therapeutic endoscopy, and prior exposure to training courses, the trainees seem well fitted to judge the simulator's realism and relevance.

The partial task training concept and the learning environment both appear to represent the intended purpose, that is, the opportunity for the rehearsal of psychomotor skills under various circumstances in a risk-free controlled environment. Second, the median global classifications of the items related to the realism of the simulator model suggests that the model mimics actual circumstances and that procedures accurately resemble elements of clinical ESD. In the survey, the wall structure of the plant-based tissue scores are respectable, but it attained the lowest value compared with other aspects of the simulator. With respect to the comparison of the model with the current animal models, a preference for the mixed-reality computer simulator is observed. This could be related to the advantage of controlling the difficulty level and unconstrained repetition that makes this model approach the ideal teaching tool. Yet, ethical and emotional considerations are likely to be also involved.

Although the findings highlight the benefits of simulation-based ESD training, this study has several limitations such as its small sample size, pilot study design, and short observation period that may not be able to depict long-term skill retention or sustained competency development with more complex interventions simulated. Also, the trainees might have rated the simulator favorably because of perceived expectations rather than actual training outcomes as an expression of survey bias. In addition, a direct comparison with a live model training is lacking.

Future Western studies should explore cost-effectiveness analyses in comparison to animal models to determine its feasibility as a large-scale training solution.

This pilot study adds to the growing evidence supporting simulation-based training for ESD. These preliminary results indicate that the EndoCubot simulator could provide a realistic, standardized, ethical and ecological alternative to animal models, demonstrating measurable improvements in trainee performance and high student satisfaction. These findings could suggest that structured simulator training can play a critical role in advanced endoscopy skill acquisition and skill maintenance. Future research should establish validated competency benchmarks for ESD training and study whether simulation-based training aids trainees in achieving these benchmarks.

## Patient consent

Because this pilot feasibility study did not involve research on patients, no local Institutional Review Board approval was needed. Only oral informed consent was obtained from all participants (health care professionals). Participants involved were free to answer the questionnaires following the conclusion of this training.

## Disclosure

All authors disclosed no financial relationships.

## Supplementary data

Video 1 Technology and capabilities of the Endocubot platform.
